# Mechanotransduction in intervertebral disc degeneration: from compartment-specific sensors to translational frontiers

**DOI:** 10.3389/fbioe.2026.1860402

**Published:** 2026-07-15

**Authors:** Hongtao Li, Changxiao Han, Guangyi Yang, Jiacheng Zheng, Bochen Peng, Jiali Chen, Hanbin Liu, Liguo Zhu, Minshan Feng

**Affiliations:** 1 Department of Spine Surgery, Wangjing Hospital, China Academy of Chinese Medical Sciences, Beijing, China; 2 Beijing Key Laboratory of Digital Intelligence Traditional Chinese Medicine for Preventing and Treating Degenerative Bone and Joint Diseases, Beijing, China

**Keywords:** cartilage endplate, intervertebral disc degeneration, mechanosensors, mechanotransduction, nucleus pulposus, Piezo1

## Abstract

Intervertebral disc degeneration is a major structural correlate of low back pain and is increasingly viewed as a disorder of mechanobiological dysregulation rather than simple mechanical overload. Over the past decade, disc cells have been shown to engage a diverse mechanosensory repertoire, including Piezo1, transient receptor potential channels, integrin–focal adhesion complexes, acid-sensing ion channels, primary cilia, and cytoskeletal–nuclear signaling axes. These upstream sensors converge on a more limited set of downstream hubs, most notably Yes-associated protein and transcriptional co-activator with PDZ-binding motif and mitogen-activated protein kinase/nuclear factor kappa B, which then shape cell-fate programs such as senescence, autophagy, pyroptosis, and ferroptosis. The extracellular matrix functions both as the substrate and as the output of mechanotransduction, creating a self-reinforcing loop in which degenerative matrix changes amplify pathological mechanosensing. Despite substantial molecular progress, mechanistic understanding remains concentrated in nucleus pulposus models, whereas matched cross-compartment analyses involving the annulus fibrosus, cartilage endplate, and vertebral interface remain limited. As a result, the relative importance of mechanosensors across compartments and loading contexts is still unresolved. Multiscale platforms, including finite element modeling, tunable hydrogels, organ culture, and quantitative magnetic resonance imaging, have strengthened causal interrogation, but translational progress remains largely preclinical. The clearest advance to date has come from biomaterial-based strategies, supported by early human feasibility data for injectable hydrogel implants. In this Review, we synthesize mechanotransduction in intervertebral disc degeneration, summarize the relative maturity of current mechanistic understanding across compartments and signaling branches, and discuss the importance of systematic cross-compartment comparison alongside single-sensor, single-compartment studies.

## 1 Introduction

Low back pain is the leading cause of years lived with disability worldwide, and intervertebral disc degeneration (IVDD) is among its most common structural correlates (Ferreira et al., 2023). The global burden of low back pain already exceeds 600 million prevalent cases and is projected to rise further with population aging and increasingly sedentary lifestyles (Ferreira et al., 2023; Chiu et al., 2025). Current treatments remain largely palliative (Chiu et al., 2025; Qiao et al., 2026). Surgical procedures such as spinal fusion address the mechanical consequences of advanced degeneration, whereas pharmacological approaches mainly alleviate current symptoms. Neither strategy restores disc biology or reverses the degenerative process (Chiu et al., 2025; Qiao et al., 2026). A deeper mechanistic understanding of IVDD is therefore essential for the development of disease-modifying therapies.

The intervertebral disc (IVD) is a mechanically specialized yet biologically constrained tissue (Qiao et al., 2026). As one of the largest avascular structures in the body, it depends on diffusion through the cartilage endplate (CEP) for nutrient supply and waste exchange, while being subjected to complex daily loading patterns that differ markedly between recumbency and upright activity (Qiao et al., 2026). Accordingly, IVDD is increasingly viewed not as a passive consequence of aging or excessive loading, but as a disorder of mechanobiological dysregulation. Nucleus pulposus (NP) cells, annulus fibrosus (AF) cells, and CEP chondrocytes detect alterations in force, stiffness, strain, and osmolarity, and convert these cues into inflammatory, metabolic, and matrix-remodeling responses (Qiao et al., 2026; Guo et al., 2023). Importantly, these processes arise within a coupled disc-endplate-vertebral unit rather than within the NP alone (Qiao et al., 2026; Yang et al., 2025).

**FIGURE 1:**
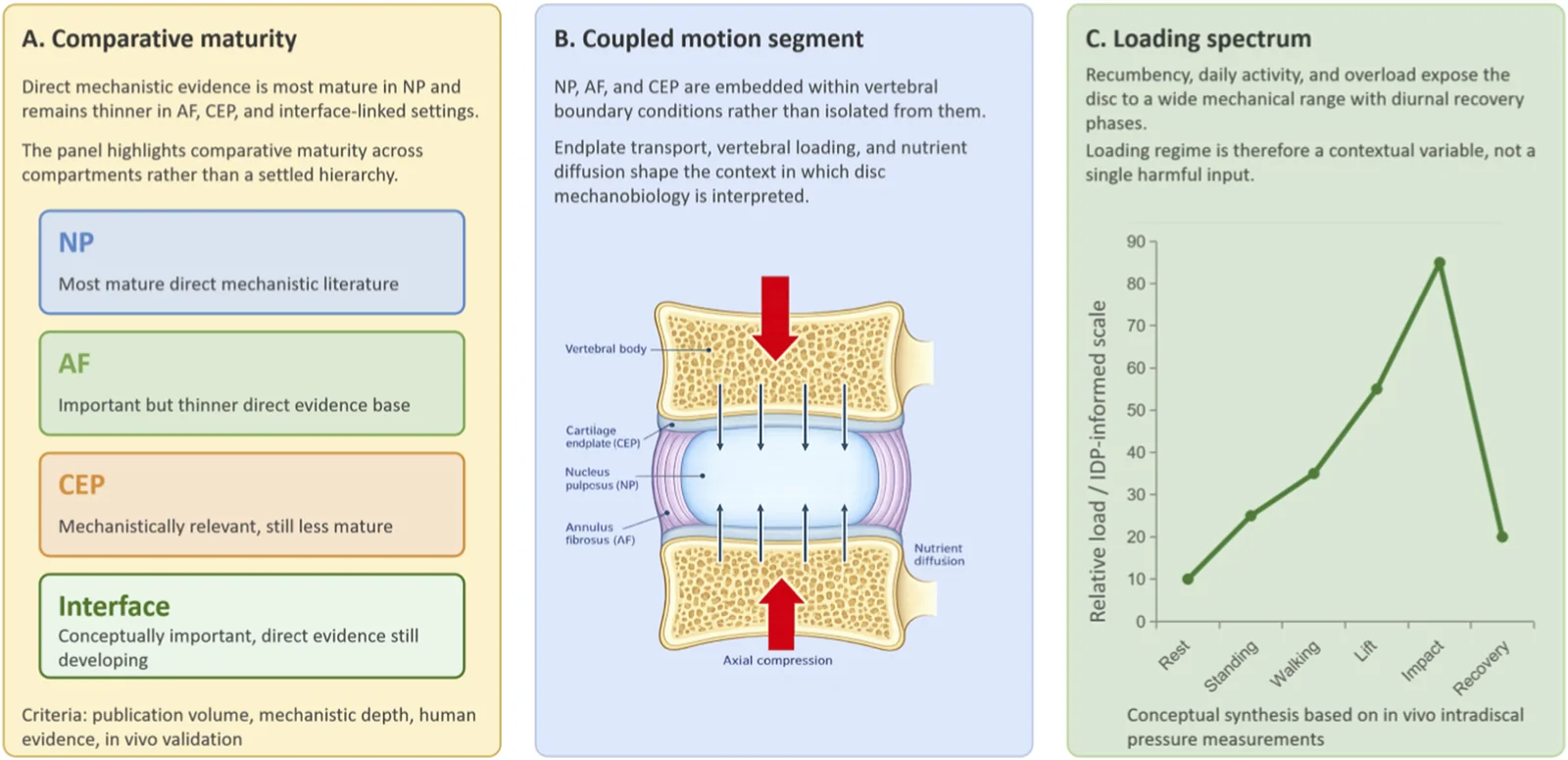
NP-predominant mechanobiology in IVDD within a disc-endplate-vertebral interface framework. **(A)** Comparative evidence maturity across NP, AF, CEP, and interface-associated mechanobiology, qualitatively ranked according to publication volume, mechanistic depth, human-tissue support, and *in vivo* validation. **(B)** Coupled motion-segment context showing that NP, AF, and CEP are mechanically and biologically constrained by vertebral boundary conditions, endplate transport, and nutrient diffusion. **(C)** Conceptual loading spectrum across recumbency, daily activity, and overload/recovery states, synthesized from reported *in vivo* intradiscal pressure measurements ranging from approximately 0.1 MPa during recumbency to above 1 MPa during upright activity and above 2 MPa during lifting (Wilke et al., 1999).

Over the past decade, the field has moved beyond descriptive associations between loading and degeneration toward a more testable mechanistic framework. The identification of Piezo1 as a mechanically gated ion channel in disc cells, the application of single-cell transcriptomics to resolve NP cell heterogeneity, and the development of tunable hydrogels and organ culture systems have each contributed to this shift (Guo et al., 2023; Yang et al., 2025; Chen S. et al., 2026; Li G. et al., 2026; Tu et al., 2022; Kang et al., 2026). However, progress across disc compartments has remained uneven. Mechanistic studies are still heavily concentrated on NP-focused models, whereas matched comparative analyses of the AF, CEP, and vertebral boundary are comparatively limited, as summarized in Figure 1A (Qiao et al., 2026; Guo et al., 2023; Chen S. et al., 2026; Li G. et al., 2026). Piezo1 is currently the most intensively studied mechanosensor in the disc (Guo et al., 2023), but whether it serves a dominant role across different compartments and loading regimes remains unresolved.

A key unresolved question is how mechanosensory pathways, downstream responses, and cell fate outcomes differ across compartments and mechanical contexts. Addressing this issue is important not only for understanding compartment-specific disease mechanisms, but also for improving the interpretation of experimental models and the design of targeted interventions. In this Review, we comprehensively analyze mechanotransduction in IVDD from an NP-centered perspective while also considering the disc-endplate-vertebral interface. We evaluate the major mechanosensors, explore common and compartment-specific downstream signaling pathways, assess the experimental platforms reshaping the field, and examine the translational implications of these findings.

## 2 Structural and cellular basis of intervertebral disc mechanobiology

The intervertebral disc is a tripartite structure comprising a central NP, a proteoglycan-rich gel that distributes compressive load, surrounded by the concentrically layered AF and capped superiorly and inferiorly by the CEP (Figure 1B) (Qiao et al., 2026; Raj, 2008). Each compartment harbors distinct cell populations with distinct mechanical set-points (Qiao et al., 2026; Kanda et al., 2021). The NP experiences predominantly hydrostatic pressure, the AF sustains tensile and shear forces, and the CEP transmits axial loads while serving as the principal route for nutrient diffusion into the avascular disc interior (Qiao et al., 2026; Osti et al., 2025).

*In vivo*, the disc experiences a complex combination of axial compression, torsion, bending, and hydrostatic pressurization, with loads fluctuating between approximately 0.1 MPa during recumbency and well above 1 MPa during upright activity or lifting (Figure 1C) (Qiao et al., 2026). Degeneration is not simply a consequence of excessive load but reflects a mismatch between the loading regime and the cellular capacity to adapt (Qiao et al., 2026). Animal experiments have demonstrated that single subfracture impacts to the endplate, without structural disruption, are sufficient to initiate a progressive IVDD phenotype through Piezo1-mediated inflammation and metabolic dysfunction (Sun et al., 2022). Conversely, chronic excessive compression drives NP cell autophagy/apoptosis imbalance via Piezo1 overexpression (Shi et al., 2022), while chronic matrix stiffening activates a Piezo1-YAP axis that promotes catabolic remodeling (Wang B. et al., 2021; Zhou et al., 2023). The ovine annular lesion model, which has contributed more than 3 decades of evidence, further demonstrates that mechanical disturbance propagates beyond the injured compartment into the CEP and vertebral trabecular bone (Osti et al., 2025). Discussions of disc mechanobiology that exclude the motion-segment bone interface may not fully capture its integrated nature (Qiao et al., 2026; Hu Z. et al., 2024). Because the CEP is the principal route for glucose, oxygen, and waste diffusion, chronic excessive endplate loading accelerates CEP degeneration (Hu Z. et al., 2024), and loss of ciliary IFT88 promotes endplate calcification under excessive stress (Dong et al., 2026). Cyclic loading combined with oxidative insults amplifies reactive oxygen species (ROS) and endoplasmic reticulum stress in NP cells (Li H. et al., 2025), and the HIF-1α/PER2/mechanistic target of rapamycin (mTOR) axis maintains autophagic rhythm under hypoxia (Yuan et al., 2025). Disc mechanotransduction is therefore shaped jointly by load, nutrition, oxygenation, and circadian timing rather than by force alone (Yuan et al., 2025; Ding et al., 2021).

Disc cell populations had long been regarded as relatively uniform groups, including NP cells, AF cells, and endplate chondrocytes. Single-cell RNA sequencing has since resolved these categories into overlapping transcriptional states and interaction patterns (Tu et al., 2022; Kang et al., 2026; Li Z. et al., 2022; Jia S. et al., 2024). Multiple human NP datasets have identified fibroblast-like, senescence-associated, progenitor-like, and notochord-remnant-like gene expression programs within the degenerating NP (Tu et al., 2022; Kang et al., 2026; Li Z. et al., 2022). Machine-learning-assisted analyses have further identified senescence-associated secretory phenotype (SASP)-high NP cell clusters that spatially associate with zones of maximal matrix catabolism and amplify inflammation in paracrine fashion (Kang et al., 2026). SPP1-centered signaling has emerged as a recurrent node linking NP-secreted osteopontin to macrophage activation, chondrocyte responses, and endplate calcification across multiple data types (Wei and Zhu, 2025; Xu et al., 2026; Li Q. et al., 2026). These datasets establish that the NP cell is a misleading singular and that mechanotransduction operates on a heterogeneous population. Therapeutic targets may ultimately need to be evaluated at the subpopulation level rather than averaged across bulk tissue. Whether these subpopulations differ in their mechanosensory properties is still unknown. For example, SASP-high senescent NP cells may express different mechanosensors from progenitor-like cells, so this heterogeneity could shape tissue-level mechanotransduction and warrants further study (Kang et al., 2026; Chen et al., 2024). One testable hypothesis is that SASP-high cells may be biased toward inflammatory and stiffness-amplified signaling, progenitor-like cells may retain more adaptive matrix-responsive programs, and fibroblast-like states may couple mechanosensing to contractility and fibrotic remodeling (Tu et al., 2022; Kang et al., 2026; Li Z. et al., 2022; Jia S. et al., 2024; Chen et al., 2024).

However, several limitations should be considered. Cell dissociation may underrepresent fibrotic NP cell states. Most human datasets also remain limited in size and heterogeneous across disease stages (Song et al., 2025). *In situ* spatial transcriptomic profiling of IVD tissue is still uncommon, and many spatial interpretations continue to rely on inference from dissociated cells rather than direct measurement (Chen et al., 2024). Likewise, bioinformatic identification of candidate genes, including ferroptosis-related regulators, should be viewed as hypothesis-generating rather than definitive mechanistic validation (Jiang et al., 2024). Species differences add a further dimension (Kanda et al., 2021; Osti et al., 2025). Rodent and porcine discs retain notochordal cells into adulthood, whereas human discs do not, implying that rescue phenotypes depending on notochordal biology may not translate directly to adult human disease (Kanda et al., 2021; Osti et al., 2025). Because key Piezo1 and Yes-associated protein/transcriptional co-activator with PDZ-binding motif (YAP/TAZ) findings come from notochordal-cell-bearing rodent models, their relevance to the notochordal-cell-depleted human disc is uncertain (Kanda et al., 2021; Osti et al., 2025). Validation in human NP cells or disc explants is therefore a priority (Kanda et al., 2021; Osti et al., 2025).

## 3 Mechanosensory receptors and upstream signaling

Disc cells experience compression, hydrostatic pressure, tensile strain, shear, osmotic cycling, and boundary-condition changes imposed by the endplate and adjacent vertebral bone (Qiao et al., 2026). The mechanosensory question is therefore inherently comparative, focusing on which receptors predominate in different compartments under distinct loading regimes (Table 1).

**TABLE 1:** Landmark studies in intervertebral disc mechanotransduction.

Year	Pathway	Key finding	Context	References
2018	Primary cilia/PTH1R-TGF-β	Ciliary PTH1R signaling maintained anabolic homeostasis during disc aging	Whole disc, NP-dominant	63
2018	IL-6/YAP1/β-catenin	IL-6/YAP1/β-catenin signaling was implicated in IVDD-associated catabolic signaling	NP	81
2019	Piezo1	Piezo1 knockdown reduced stretch-induced mitochondrial dysfunction and apoptosis in nucleus pulposus cells	NP	31
2020	TRPV4	TRPV4 mediated hyperphysiological stretch-induced inflammatory signaling; CRISPR-Cas9 knockout attenuated this response	AF	42
2020	Integrin α5β1	Integrin α5β1 was required for maintenance of notochordal cells under dynamic loading	NP/whole-disc loading context	12
2020	YAP pharmacologic modulation	Verteporfin restored rounded phenotype and broad gene-expression features in human NP cells	NP	82
2021	Piezo1	Matrix stiffness activated Piezo1 and promoted oxidative stress-associated senescence/apoptosis	NP	16
2022	Piezo1/endplate impact injury	Single impact injury of vertebral endplates initiated IVDD through Piezo1-associated inflammation and metabolic dysfunction	Endplate-disc interface	14
2022	Piezo1/NF-κB/periostin	A self-amplifying Piezo1-NF-κB-periostin loop accelerated mechano-induced NP cell senescence	NP	37
2023	Piezo1/Drp1	Matrix stiffness induced Drp1-mediated mitochondrial fission through Piezo1 mechanotransduction	NP	38
2023	YAP/TEAD1	Matrix stiffness activated YAP/TEAD1-Cyclin B1 signaling linked to proliferation-associated degeneration	NP	17
2024	Piezo1/ferroptosis	Piezo1 mediated mechanical stress-associated iron influx and exaggerated ferroptosis in NP cells	NP	34
2024	TRPV4	Conditional Trpv4 deletion protected the disc from load-induced degeneration and preserved ECM composition	Whole disc/cartilaginous disc tissues	40
2025	Piezo1	Disc-targeted Piezo1 deletion alleviated age- and stress-associated IVDD	Whole disc	33
2025	Matrix-matched biomaterial	One-year follow-up of an injectable hydrogel implant reported feasibility and acceptable safety in painful lumbar DDD	Translational/human feasibility	144
2026	Piezo1/YAP-TEAD/NLRP3	Compressive stress induced cartilage endplate degeneration through a Piezo1/YAP-TEAD/NLRP3 axis	CEP	35
2026	Piezo1/BMP2 ossification	Piezo1 suppressed BMP2-induced ossification in AF cells, suggesting compartment-specific and non-uniform effects	AF	72
2026	Matrix viscous dissipation	An engineered hydrogel identified matrix viscous dissipation as a disease-relevant mechanical variable linked to IVDD severity	Matrix mechanics/translational platform	116

### 3.1 Piezo channels

Piezo1 entered the IVDD field through human NP cell studies linking mechanical stretch to mitochondrial dysfunction and apoptosis (Yang Q. et al., 2019). Subsequent work extended this branch to stiffness-driven senescence and apoptosis in human NP cells, endplate impact injury in rat discs, chronic excessive compression associated with Piezo1 overexpression and NP cell autophagy/apoptosis imbalance, and AF cell apoptosis under aberrant loading (Sun et al., 2022; Shi et al., 2022; Wang B. et al., 2021; Liu et al., 2023). The most compelling *in vivo* findings came from disc-specific Piezo1 deletion in inducible aggrecan-CreERT2 mice, which reduced age- and load-associated degeneration (Li F. et al., 2025). However, this model does not resolve NP, AF, and CEP contributions separately, and the accompanying human data are association-level rather than functional.

**FIGURE 2:**
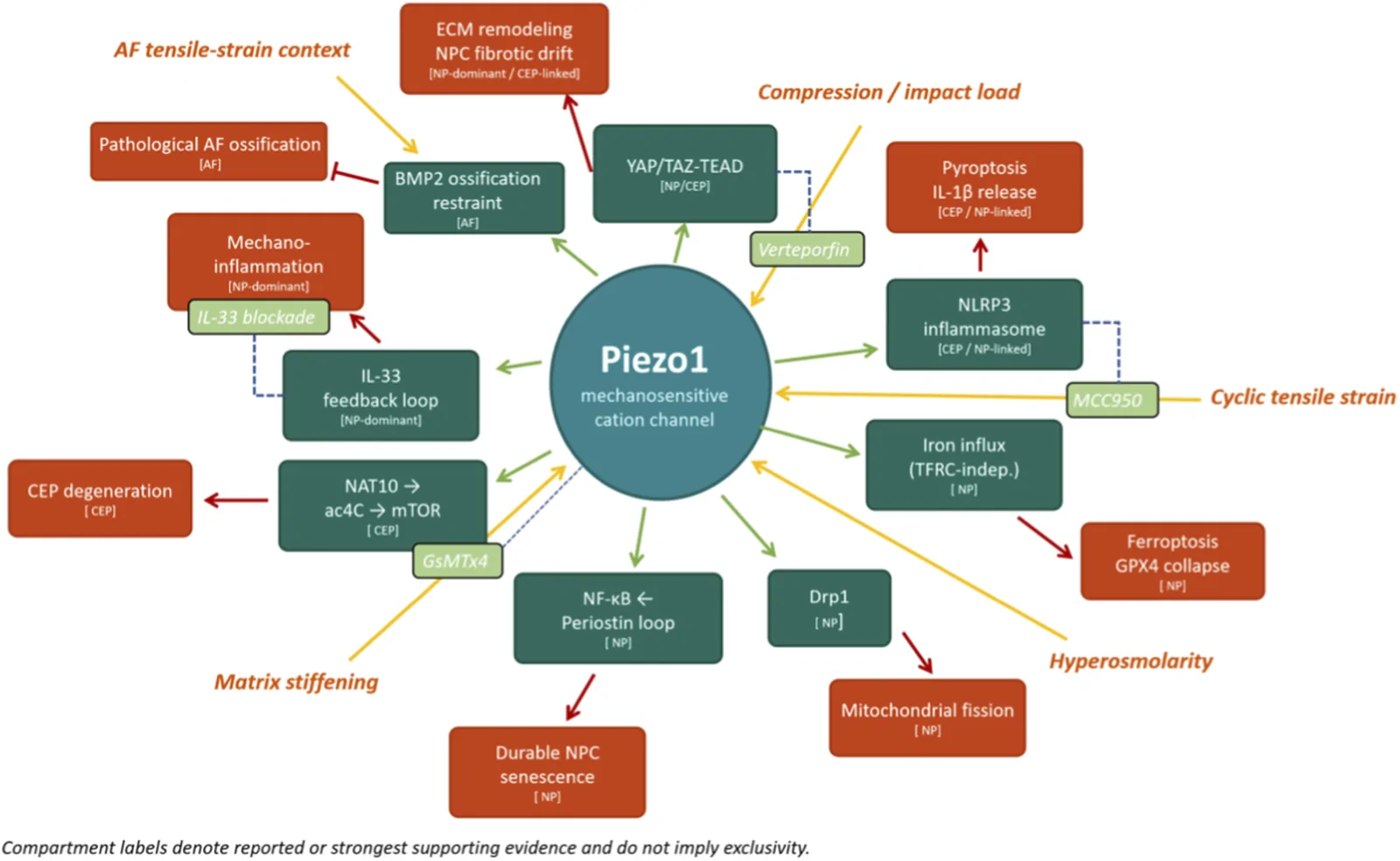
Piezo1-associated mechanotransduction network in IVDD. Mechanical inputs, including compression or impact load, cyclic tensile strain, hyperosmolarity, matrix stiffening, and AF tensile-strain context, converge on Piezo1-related pathological or context-dependent modules. These branches include YAP/TAZ-TEAD signaling, NLRP3-mediated pyroptosis, iron-driven ferroptosis, Drp1-mediated mitochondrial fission, NF-κB/periostin-associated senescence, NAT10/ac4C/mTOR signaling in the CEP, IL-33 feedback, and AF-related BMP2 ossification restraint. Compartment labels denote reported or strongest supporting evidence and do not imply exclusivity.

Multiple downstream branches have been linked to Piezo1 in disc cells (Figure 2). These include YAP/TAZ-TEAD transcriptional activation, NLR family pyrin domain containing 3 (NLRP3) inflammasome-dependent pyroptosis, and transferrin-receptor-independent iron influx leading to ferroptosis (Yang et al., 2025; Zhou et al., 2023; Xiang et al., 2024; Cao et al., 2026; Zhuang et al., 2025). Additional pathways involve Drp1-mediated mitochondrial fission, a self-amplifying nuclear factor kappa B (NF-κB)/periostin senescence loop, and NAT10/ac4C/mTOR signaling in CEP chondrocytes (Wu et al., 2022; Ke et al., 2023a; Sun et al., 2026). Importantly, the current level of mechanistic characterization varies substantially across these pathways. YAP activation and NLRP3-mediated pyroptosis have been described across multiple model systems, whereas iron-influx-driven ferroptosis and the CEP-specific NAT10 branch have so far been examined in more limited settings and await broader validation.

At present, two issues warrant caution when interpreting Piezo1 as a central mechanosensor. First, to our knowledge, no study has systematically compared Piezo1 dependence across NP, AF, and CEP using a directly matched design (Sun et al., 2022; Shi et al., 2022; Wang B. et al., 2021; Zhou et al., 2023; Liu et al., 2023; Li F. et al., 2025; Xiang et al., 2024; Cao et al., 2026; Zhuang et al., 2025; Wu et al., 2022; Ke et al., 2023a; Sun et al., 2026). Most available data derive from compartment-specific experiments, and quantitative dose-response relationships for Piezo1 activation in each cell type remain to be defined (Sun et al., 2022; Shi et al., 2022; Wang B. et al., 2021; Zhou et al., 2023; Liu et al., 2023; Li F. et al., 2025; Xiang et al., 2024; Cao et al., 2026; Zhuang et al., 2025; Wu et al., 2022; Ke et al., 2023a; Sun et al., 2026). Second, Piezo2 has no comparable body of disc-cell-focused evidence and has not been systematically examined across NP, AF, and CEP cells. Accordingly, current understanding is derived largely from studies of Piezo1, and Piezo2 remains largely unexamined in disc cells.

### 3.2 Transient receptor potential channels and osmosensing

Transient receptor potential (TRP) channels constitute the second major family of mechanosensitive ion channels in the disc, with transient receptor potential vanilloid 4 (TRPV4) as the best characterized member (Kim et al., 2024; Gan et al., 2018). Low-magnitude cyclic compression enhances mesenchymal stem cell biosynthesis toward an NP-like phenotype via a TRPV4-dependent pathway (Gan et al., 2018). Hyperphysiological cyclic stretch activates TRPV4 in human AF cells and drives an inflammatory program that TRPV4 inhibition or CRISPR-Cas9 knockout abolishes (Cambria et al., 2020). Hyperphysiological compression of bovine NP cells activates TRPV4 upstream of COX2/PGE2, directly linking the channel to prostaglandin-mediated signaling (Cambria et al., 2021). In intact bovine IVD organ culture, dynamic modulation of TRPV4 protects against sustained-loading degeneration and promotes matrix synthesis, indicating that TRPV4 function is regime-dependent rather than uniformly protective or pathogenic (Easson et al., 2023). The *in vivo* relevance of TRPV4 was confirmed by a conditional Trpv4 knockout in Col2-Cre; Trpv4fl/fl mice, which were protected from load-induced IVDD with preserved extracellular matrix (ECM) composition (Kim et al., 2024).

TRPV4 is not the sole osmosensor of the NP. Hypoosmotic loading of NP cells induces IL-6 independently of both TRPV4 and TRPM7, pointing to additional, still uncharacterized osmosensing routes (Sadowska et al., 2020). TRPV4 also participates in a wider osmoadaptive network alongside aquaporins one and 4, which together govern NP cell volume under diurnally fluctuating hyperosmolarity (Snuggs et al., 2021). Beyond TRPV4, matrix stiffness has been linked to TRPV2-dependent NP cell apoptosis through cytoskeletal reorganization (Yu et al., 2021), and TRPA1 is induced by inflammatory cytokines in degenerated disc cells and linked to altered IL-8 and matrix-gene expression (Kameda et al., 2019). The interplay between TRP channels and Piezo1, and their relative contributions under matched assay conditions, remain to be clarified.

### 3.3 Integrin and focal adhesion signaling

Integrin-mediated adhesion to the ECM constitutes the second major arm of disc mechanotransduction alongside ion-channel sensing (Kanda et al., 2021; Zhan et al., 2026; Kurakawa et al., 2015). Integrin α5β1 is among the best characterized mechanoreceptors in disc cells (Kanda et al., 2021; Kurakawa et al., 2015). In *ex vivo* dynamic-loading experiments, α5β1 is required for the normal maintenance of notochordal cells under pathological compression, and its disruption accelerates notochordal disappearance (Kanda et al., 2021). Region-dependent analysis has shown that α5β1 is differentially expressed across NP, AF, and CEP and that its role in balancing autophagy and apoptosis is compartment-specific (Zhan et al., 2026). On stiff substrates, NP cells undergo integrin β1-p38-MAPK (mitogen-activated protein kinase)-driven senescence, and exogenous lysyl oxidase (LOX) partially rescues this phenotype by remodeling matrix crosslinks (Zhao et al., 2022).

The focal adhesion complex contributes several additional mechanosensitive nodes. Kindlin-2, a focal adhesion adaptor highly expressed in the NP, inhibits NLRP3 inflammasome activation and is required to maintain disc homeostasis (Chen S. et al., 2022). Caveolin-1 has been linked to AF mechanobiology, where moderate mechanical stimulation reduces Cav1-related proinflammatory signaling (Zhang W. et al., 2021), and substrate topography regulates AF-derived stem cell differentiation through a CAV1-YAP axis (Chu et al., 2021; Chu et al., 2019). SPP1 (osteopontin) secreted by stressed NP cells engages integrin α5β1 on neighboring cells to inhibit mitophagy through PINK1/PARKIN blockade, accelerating NP calcification (Gu et al., 2025). Fibronectin-peptide-functionalized hydrogels activate a contractile phenotype in NP cells (Naha et al., 2025), and fluid shear stress acting through integrin-coupled signaling regulates HO-1 expression, autophagy, and ECM homeostasis (Chen et al., 2020). The focal adhesion complex may therefore be better viewed not as a single pathway, but as a network with multiple nonredundant entry points.

### 3.4 Acid-sensing ion channels

The disc interior is chronically hypoxic and acidic, with lactate accumulating progressively during degeneration (Zhou et al., 2016; Zhao et al., 2021; Liu et al., 2025). Acid-sensing ion channels (ASICs), long implicated in nociceptive signaling in degenerative disease (Zhou et al., 2016), are therefore natural candidate degenerative mechanosensors. ASIC1/ASIC3 activation by lactate-driven acidification in NP cells drives NLRP3 inflammasome-dependent pyroptosis, linking the disc’s acidic microenvironment directly to inflammatory cell death (Zhao et al., 2021). ASIC3 has further been shown to contribute to the elongation of NP cells under mechanical loading, a morphological change that correlates with degeneration severity (Lim et al., 2024). This places ASIC3 in a dual position as both an acid sensor and a mechanical responder, and suggests that ASIC activation may be related to Piezo1-driven NLRP3 signaling through a shared inflammasome effector node.

### 3.5 Primary cilia and cytoskeletal mechanics

Primary cilia are microtubule-based organelles that project from the cell surface into the pericellular matrix and function as mechanosensors in connective tissues (Zheng et al., 2018; Li X. et al., 2022). In the disc, NP cell primary cilia transport parathyroid hormone 1 receptor (PTH1R) to the ciliary membrane under mechanical stress, and ciliary PTH signaling is required to maintain transforming growth factor-β (TGF-β)-dependent anabolic activity in aging discs (Zheng et al., 2018). Conditional deletion of the intraflagellar transport protein IFT80 produces severe defects in IVD development and maintenance (Li et al., 2020). NP primary cilia change their length in response to extracellular osmolarity, suggesting a cilia-linked osmosensory adaptation partly separable from TonEBP-mediated osmoregulation (Choi et al., 2019). In endplate chondrocytes, ciliary IFT88 inhibits IVDD under excessive mechanical stress by restraining endplate cartilage calcification (Dong et al., 2026). Broader reviews now place primary cilia among the core mechanosensors of the musculoskeletal system alongside Piezo1 and integrin-focal-adhesion complexes (Ti et al., 2025).

The cell interior itself functions as a mechanical organ (Yu et al., 2021; Song et al., 2022). Matrix-stiffness information is relayed from surface receptors to the nucleus through the actin cytoskeleton and its regulatory machinery. ECM stiffness drives TRPV2-dependent NP cell apoptosis through actin cytoskeleton reorganization (Yu et al., 2021). Inhibition of the RhoA/myocardin-related transcription factor A (MRTF-A) axis alleviates NP fibrosis induced by mechanical stress overload, and matrix stiffness drives changes in NP cell glycolysis through MRTF-A-dependent signaling (Song et al., 2022; Xu et al., 2025). Together, these studies identify RhoA/MRTF-A as a mechanotransduction hub operating in parallel with YAP/TAZ, with both modules being actin-cytoskeleton-sensitive transcriptional integrators activated by matrix stiffening. The Hippo pathway further orchestrates cytoskeletal organization during IVDD, and microtubule stabilization promotes type II collagen synthesis in NP cells by activating Hippo-YAP signaling, reinforcing the bidirectional coupling between cytoskeletal state and YAP/TAZ activity (Zhang C. et al., 2021; Zhang et al., 2023). Additionally, nuclear mechanics, including the role of Lamin A/C and LINC complexes in translating nuclear shape into chromatin state, remain underexplored in the disc and represent a clear priority for future work.

### 3.6 Toward a comparative mechanosensor framework

The disc possesses a diverse repertoire of mechanosensors, although current mechanistic understanding remains centered largely on Piezo1 and NP models (Table 2) (Wang B. et al., 2021). A central unresolved question is not simply whether Piezo1 is involved, but how the relative importance of mechanosensors may vary across compartments and interfaces. Directly matched experiments using comparable cell types, mechanical stimuli, and readouts could help determine whether TRPV4, integrin α5β1, or primary-cilium signaling produces effects comparable to or greater than those associated with Piezo1 perturbation (Cambria et al., 2020). Quantitative studies showing that CEP chondrocytes, AF cells, or interface-coupled motion-segment models preferentially transduce dominant mechanical cues through non-Piezo1 sensors would indicate a compartment-dependent hierarchy (Liu et al., 2023). Conditional Piezo1 gain-of-function experiments, together with additional AF-related protective observations such as the reported suppression of bone morphogenetic protein 2 (BMP2)-induced ossification by Piezo1 in AF cells, would support a regime- and compartment-specific view of signaling rather than a uniformly degenerative model (Shitozawa et al., 2026). Although disc-specific Piezo1 deletion in mice supports biological plausibility, its relevance to human disc tissues remains to be established, highlighting the need for matched human *ex vivo* validation before direct rodent-to-human extrapolation (Li F. et al., 2025). At present, these questions have not been addressed in a systematic cross-compartment manner. Taken together, current findings place Piezo1 among the leading candidate mechanosensors, while additional cross-compartment studies will help clarify how its role varies across cellular and biomechanical contexts. Each compartment is tuned to a different mechanical regime, with hydrostatic pressure in the NP, tensile and shear strain in the AF, and compressive load in the CEP (Qiao et al., 2026). Resident cells likely calibrate their mechanosensors to this regime, which helps explain why Piezo1 can be catabolic in the NP yet protective in the AF and why uniform, disc-wide targeting may be ineffective (Wang B. et al., 2021; Zhou et al., 2023; Shitozawa et al., 2026). Based on current compartment-specific evidence, NP cells are expected to prioritize osmotic, hydrostatic-pressure, and volume-regulatory sensing; AF cells are more likely to weight integrin-cytoskeletal and tensile-strain pathways; and CEP chondrocytes may preferentially engage compression-linked inflammatory, nutrient-transport, and calcification programs (Qiao et al., 2026; Kanda et al., 2021; Osti et al., 2025; Wang B. et al., 2021; Zhou et al., 2023; Liu et al., 2023; Cambria et al., 2020; Shitozawa et al., 2026). These differences imply that the same molecular node can occupy different positions in the local signaling hierarchy depending on native matrix architecture and loading mode (Qiao et al., 2026; Wang B. et al., 2021; Zhou et al., 2023; Shitozawa et al., 2026). Table 3 summarizes these compartment-specific differences across loading environment, mechanosensors, downstream signaling, and biological outcome.

**TABLE 2:** Comparative overview of candidate mechanosensors across disc compartments and interface contexts.

Mechanosensor	NP	AF	CEP	Interface context
Piezo1	Most developed across models	Limited and mixed; possible protective role	Compression-, pyroptosis-, and mTOR-related links	Likely relevant; direct studies sparse
TRPV4	Recurrent *in vitro* and organ-culture findings	Limited stretch-related findings	Limited direct characterization	Very limited direct data
Integrin/focal adhesion	Recurrent links to stiffness sensing and fibrosis	AF survival and stretch responses described	Limited direct characterization	Mostly inferred from coupling models
ASIC	Recurrent acid- and lactate-linked stress responses	Sparse	Limited inflammasome-related links	Minimal direct data
Primary cilia	Mechanically responsive and protective signaling reported	Few direct studies	Limited CEP/endplate links	Likely relevant; direct characterization limited
Cytoskeletal-nuclear axis	Growing characterization of stiffness and nuclear mechanics	Conceptually relevant but incompletely mapped	Sparse direct data	Mostly indirect

**TABLE 3:** Cross-compartment synthesis of disc mechanobiology.

Compartment	Loading	Key sensors	Key signaling	Main outcomes	Maturity
Nucleus pulposus	Hydrostatic, osmotic	Piezo1, TRPV4, ASIC, cilia	YAP/TAZ, NF-κB, mitochondrial	Senescence, pyroptosis, ferroptosis	High
Annulus fibrosus	Tensile, shear	Integrins, stretch channels, Piezo1	Integrin-survival, NF-κB	Apoptosis, fibrosis, ossification restraint	Moderate
Cartilage endplate	Compression, transport	Piezo1, cilia, TRPV4	YAP/TEAD/NLRP3, mTOR	Calcification, pyroptosis	Low
Disc-endplate-vertebral interface	Coupled transfer	Largely inferred	Cross-compartment spread	Endplate/bone remodeling	Very low

### 3.7 Physiological and homeostatic mechanotransduction

Dysregulation is best read against normal regulation. Physiological cues sustain disc homeostasis through TRPV4-mediated matrix maintenance (Cambria et al., 2020; Cambria et al., 2021; Easson et al., 2023), ciliary PTH1R and TGF-β signaling (Zheng et al., 2018), Kindlin-2 restraint of NLRP3 (Chen S. et al., 2022), physiological hydrostatic pressure (Wang Y. et al., 2021; Vieira et al., 2021), moderate mechanical stimulation (Zhang W. et al., 2021; Wang H. et al., 2023), ERK1/2 survival signaling (Ge et al., 2019), and protective autophagy (Yang M. et al., 2019; Yurube et al., 2021). Degeneration can therefore be viewed as a shift away from this healthy operating window rather than only as the gain of pathological signaling.

## 4 Downstream signaling networks and cell fate decisions

**FIGURE 3:**
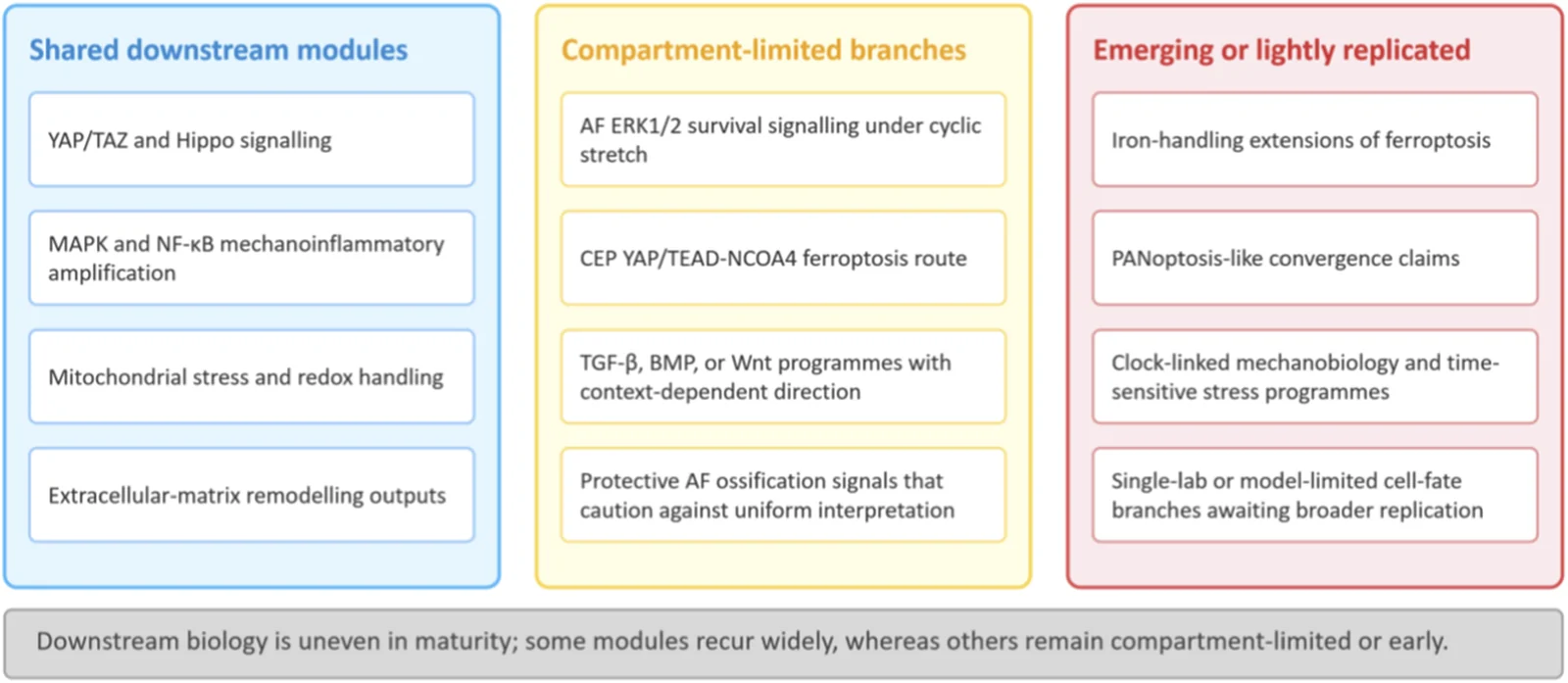
Hierarchy of mechanotransduction modules in IVDD. Shared modules (blue) feature well-established pathways like YAP/TAZ and NF-κB common across disc tissues; compartment-limited branches (yellow) highlight site-specific responses such as AF-protective ossification and CEP ferroptosis; and emerging mechanisms (red) outline novel frontiers like clock-linked mechanobiology and PANoptosis. The bottom banner (gray) advocates for a compartment-sensitive interpretation rather than a uniform model of mechanical stress across the intervertebral disc.

**FIGURE 4:**
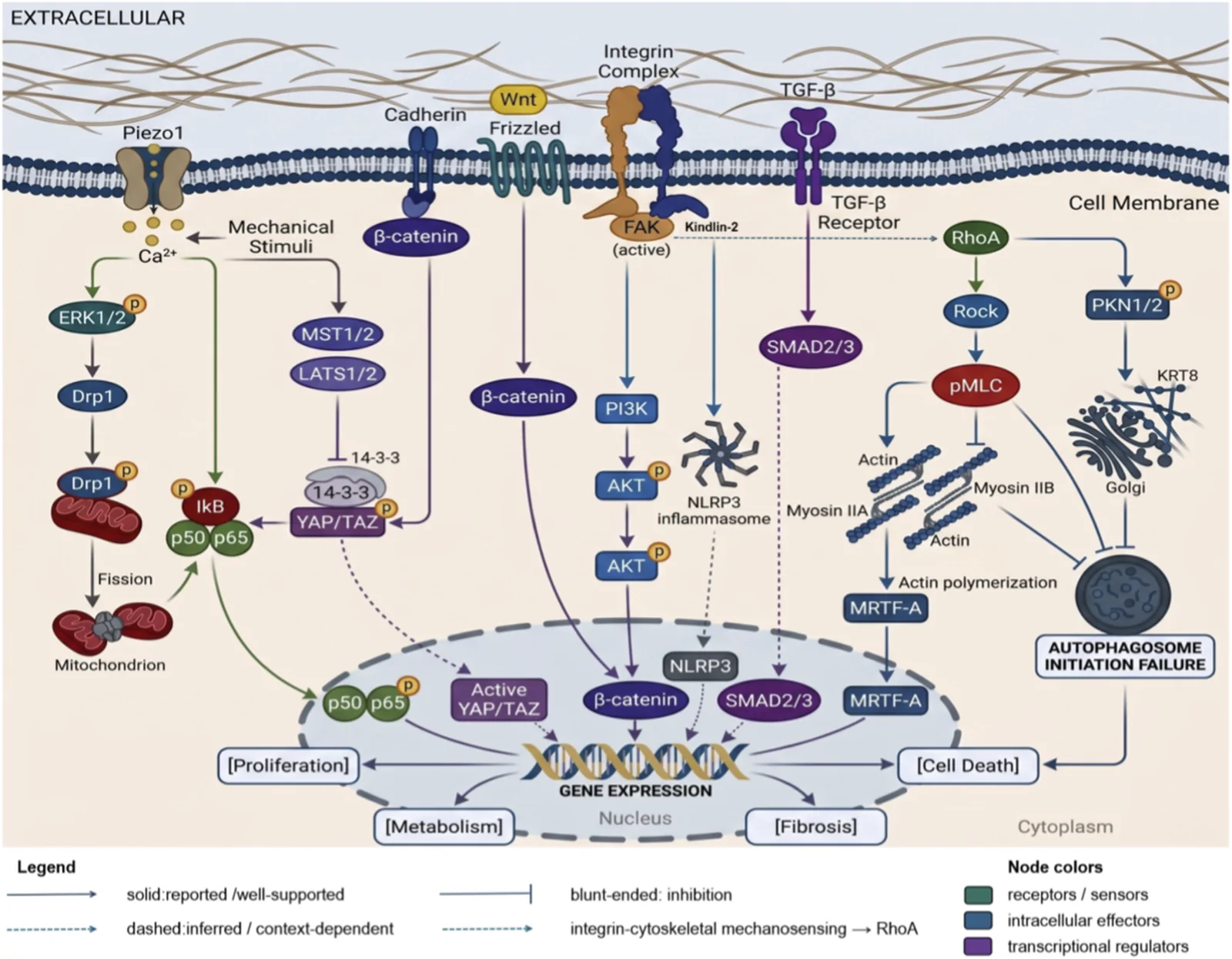
Cellular microenvironment sensing and multi-receptor signaling network. This diagram illustrates the molecular mechanisms by which cells sense extracellular mechanical forces and biochemical signals via membrane receptors such as Piezo1, integrins, Wnt, and TGF-β. Upon activation, these receptors transduce external stimuli into multiple intracellular signaling cascades involving mitochondrial dynamics, cytoskeletal remodeling, and kinase pathways. These signals ultimately drive key transcription factors into the nucleus to regulate gene expression, thereby determining cellular fates such as proliferation, metabolism, fibrosis, or cell death. The RhoA and MRTF-A branch is driven by integrin and cytoskeletal mechanosensing and is shown explicitly. Node colors denote membrane receptors, intracellular effectors, and transcription factors, arrow colors denote activation versus inhibition, and line styles denote direct versus inferred connections.

Whether an intervertebral disc cell survives, senesces, transdifferentiates, or undergoes inflammatory death depends on which downstream networks are engaged (Figure 3; Table 4). Across compartments, distinct mechanosensors converge on a small set of shared hubs, including YAP/TAZ, NF-κB, MAPK, mTOR, and MRTF-A, which then select cell-fate programs that remodel the matrix and ultimately drive clinical disc degeneration (Yuan et al., 2025; Wu et al., 2022; Ke et al., 2023a; Sun et al., 2026; Kurakawa et al., 2015; Song et al., 2022; Wang H. et al., 2023). Figure 4 summarizes this sensor-to-matrix axis and serves as the integrative backbone for the analyses that follow. Several of these hubs are additionally gated by circadian timing (Yuan et al., 2025; Ding et al., 2021).

**TABLE 4:** Major mechanotransduction pathways in IVDD and their current limitations.

Branch	Main upstream inputs	Human validation	Key downstream outputs	Main limitation
Piezo1-centered mechanotransduction	Matrix stiffness, compression, impact loading, mechanical overload, Ca^2+^ influx	Human tissue upregulation and human primary-cell perturbation	YAP/TAZ activation, NF-κB/periostin amplification, senescence, catabolic remodeling, mechanotranscriptional activation	Cross-compartment ranking remains unresolved; AF/CEP contributions are not cleanly separated in most models
TRPV4-associated mechanotransduction	Stretch, compression, osmotic load	Human NP cell support	Inflammation, COX2/PGE2 signaling, ECM remodeling	Direct CEP characterization remains limited; head-to-head comparison with Piezo1 is lacking
Integrin/focal adhesion signaling	Matrix adhesion, stiffness, dynamic loading	Human AF and NP primary-cell support	Survival signaling, fibrosis, senescence, autophagy/mitophagy regulation, MAPK/NF-κB coupling	CEP-specific direct mechanistic characterization is thinner than for NP/AF
Primary cilia signaling	Mechanical stress, osmotic change, aging-associated loading	Human cell support present	Anabolic maintenance, osmoadaptation, endplate calcification control	AF characterization is sparse; direct comparative studies with ion channels are lacking
ASIC-linked stress signaling	Acidification, lactate accumulation, mechanical loading	Mainly indirect or descriptive in human tissue/cells	NLRP3 activation, pyroptosis, mechanosensitive elongation	More limited than Piezo1, TRPV4, or integrin pathways
Inflammatory and degenerative execution branches	Piezo1, TRPV4, integrins, ASICs, oxidative stress, iron handling, ROS, matrix stiffening	Limited direct human validation	MAPK/NF-κB activation, pyroptosis, ferroptosis, mitochondrial dysfunction, ECM degradation	Compartment specificity and upstream hierarchy remain incompletely resolved

Human validation here is graded by increasing strength, namely, human tissue association, human primary-cell perturbation, human *ex vivo* validation, and clinical evidence, and each entry shows the strongest level reported.

### 4.1 YAP/TAZ as a convergent mechanotranscriptional hub

YAP/TAZ integrates inputs from matrix stiffness, cytoskeletal tension, adhesion state, and membrane mechanics, making it one of the most recurrent downstream modules in disc mechanobiology (Panciera et al., 2017; Totaro et al., 2018). Support is strongest in tunable-stiffness NP systems, where substrate stiffness activates YAP/TEAD and promotes catabolic remodeling (Zhou et al., 2023). Additional studies place YAP downstream of IL-6 signaling (Chen et al., 2019), demonstrate that Verteporfin rescues human NP cell morphology (Fearing et al., 2020), show that irisin activates LATS/YAP/CTGF signaling (Chen T. et al., 2022), and link hydrostatic-pressure regimes to Hippo signaling (Wang Y. et al., 2021; Vieira et al., 2021). However, the recurrence of YAP should not be mistaken for uniform upstream control. Some YAP entries are Piezo1-associated, whereas others are clearly Piezo1-independent, and the balance appears compartment- and regime-specific. Non-Piezo1 YAP entries sharpen this point. AF cells show YAP-mediated suppression of NF-κB under moderate cyclic stimulation (Wang H. et al., 2023). Fibronectin ligation activates YAP and contractility in NP cells (Naha et al., 2024). IL-32 disturbs metabolism through FAT4-mediated Hippo-YAP signaling (Li et al., 2024). SEPHS1 delays NP cell senescence through Hippo-YAP/TAZ signaling (Hu T. et al., 2024). CEP chondrocytes enter ferroptosis through a YAP/TEAD1/NCOA4 route (Wang et al., 2025). YAP is therefore better regarded as a convergent downstream hub than as a marker of Piezo1 primacy, but current support is still largely preclinical, predominantly NP-focused, and comparatively limited for the AF and CEP.

### 4.2 MAPK/NF-κB inflammatory amplification

If YAP/TAZ is the transcriptional integrator of mechanical input, MAPK and NF-κB are the dominant inflammatory amplifiers. NF-κB mediates convergence of inflammation, oxidative stress, and mechanical stress to drive NP cell dysfunction and ECM degradation (Zhang GZ. et al., 2021). The p38-MAPK arm is linked to the integrin-β1/p38-MAPK axis in stiffness sensing, through which NP cells on stiff substrates enter senescence (Zhao et al., 2022). The ERK1/2 arm is protective rather than pathological in AF cells subjected to cyclic stretch, where β1 integrin inhibits stretch-induced apoptosis through ERK1/2 (Zhang et al., 2016). This divergence underscores that MAPK outcomes depend sharply on the isoform engaged and the cell type involved. Downstream of these kinase arms, a self-amplifying NF-κB/periostin loop initiated by Piezo1 converts a single mechanical insult into durable NP cell senescence (Wu et al., 2022). Natural-product pharmacology has repeatedly nominated MAPK/NF-κB as a tractable node, with glycitin suppressing inflammation and oxidative stress via this axis (Zhao et al., 2023) and miR-155-5p modulating mechanical-load-dependent inflammatory output (Cazzanelli et al., 2024). The mechanoinflammatory output of the disc is thus not dominated by any single kinase but reflects a balance between ERK1/2-mediated survival and p38-MAPK/NF-κB-mediated catabolism, with integrin β1 sitting immediately upstream of this balance.

### 4.3 Reactivation of developmental pathways

Classical developmental pathways are reactivated in degenerating discs, and recent data demonstrate that their engagement is partly mechanical (Zheng et al., 2018; Fu et al., 2021; Li et al., 2025c). Aberrant spinal mechanical loading has been linked to TGF-β-associated pyroptosis and nerve ingrowth into the disc (Fu et al., 2021). The ciliary PTH1R/TGF-β axis operates on the protective side of the same pathway, illustrating that TGF-β signaling can be either anabolic or catabolic depending on the upstream mechanosensor and cellular context (Zheng et al., 2018). Exosome-delivered SKI mRNA, an endogenous TGF-β repressor, reverses NP fibrosis in degenerated discs, providing the first RNA-based therapeutic interrogation of TGF-β overactivation in IVDD (Li et al., 2025c).

Wnt signaling is emerging with comparable mechanical coupling. Single-cell RNA sequencing of human degenerative NP cells has identified a Wnt/Ca^2+^ branch enriched in apoptotic and inflammatory subpopulations (Wang P. et al., 2024). Non-canonical PKC-mediated Wnt/β-catenin signaling regulates MMP expression in NP cells and intersects with senescence (Quan and Kim, 2024). BMP signaling warrants particular attention. A 2026 AF study suggests that Piezo1 restrains BMP2-induced ossification, directly countering any simple narrative that Piezo1 is uniformly harmful (Shitozawa et al., 2026). This argues against disc-wide Piezo1 blockade as a uniform strategy. More broadly, the current TGF-β, BMP, and Wnt literature still draws on a mixture of correlative observations, single-cell-based inferences, and perturbation studies, and the disc-specific hierarchy among these pathways has yet to be clearly established.

Mechanistically, loss of inhibitory SMAD control raises TGF-β and BMP activity and drives annulus fibrosus ossification that is reversible by pathway modulation (Zieba et al., 2022), whereas endplate defects that alter local mechanics raise BMP-2 and promote calcification (Imran et al., 2025). TGF-β also sustains nucleus pulposus fibrosis through RhoA and MRTF-A (Song et al., 2022; Li et al., 2025c), and overloaded axial stress activates Wnt3a and β-catenin in human NP cells to promote apoptosis (Cai et al., 2023), so these developmental pathways are compartment-dependent rather than uniformly degenerative. Developmental signaling further gates disc vascularity. Vascular endothelial growth factor (VEGF) is present in healthy nucleus pulposus but is restrained by the avascular aggrecan-rich matrix (Johnson et al., 2005), and during degeneration aggrecan loss with IL-1β driven VEGF and nerve growth factor promotes neurovascular ingrowth (Binch et al., 2014), while mechanical load itself regulates endplate VEGFA and vascular buds (Zhan et al., 2021).

### 4.4 Cell death modalities in mechanically stressed discs

Mechanotransduction does not produce a single death signal but biases disc cells toward a spectrum of stereotyped fates (Ke et al., 2025). The choice among these fates appears to be governed by thresholds of Ca^2+^ influx, ROS levels, iron load, mitochondrial dynamics, and YAP activity, although the ordering of these thresholds is still more often inferred than directly dissected (Ke et al., 2025; Zhang W. et al., 2022).

Apoptosis and senescence represent the most extensively studied endpoints (Kang et al., 2026; Wu et al., 2022; Zhao et al., 2022). Compression-induced oxidative stress damage to NP cells can be ameliorated by Bardoxolone methyl, which targets the Nrf2/ARE axis upstream of apoptosis and ECM degradation (Tian et al., 2022). Under cyclic mechanical tension, autophagy acts as a protective response against NP cell apoptosis, and static loading models similarly implicate autophagy in preserving notochordal cell survival (Yang M. et al., 2019; Yurube et al., 2021).

Senescence is reached through multiple upstream routes, including Piezo1/NF-κB/periostin, integrin-β1/p38-MAPK, and Hippo-YAP-associated branches, each of which has been linked to SASP-high NP cell states (Kang et al., 2026; Wu et al., 2022; Zhao et al., 2022; Hu T. et al., 2024). mTOR remains an important autophagy-governing kinase in disc models, including hypoxia-associated autophagic regulation and CEP responses under excessive compression (Yuan et al., 2025; Sun et al., 2026). Non-coding RNA layers add complexity, exemplified by circRNA hsa_circ_0101645 sponging miR-1304-5p to regulate BNIP3 expression and drive NP cells toward excessive autophagy and apoptosis (Luo et al., 2026). YAP1 has also been implicated in aggravating NP cell pyroptosis and senescence by promoting BNIP3-mediated mitophagy (Peng et al., 2024).

Pyroptosis represents one of the more clearly defined inflammatory death branches (Cao et al., 2026; Zhao et al., 2021). Mechanically inducible in the disc, it is engaged through multiple entry routes, including ASIC-linked acid sensing, direct Piezo1-related Ca^2+^ signaling, and the CEP Piezo1/YAP-TEAD/NLRP3 branch (Cao et al., 2026; Zhao et al., 2021). Ferroptosis, although expanding rapidly, remains less mature. Stiffness-driven YAP/N-cadherin ferroptosis in NP cells, Piezo1-associated iron influx, CRISPLD2-mediated suppression of ferroptosis, and macrophage-derived legumain modulation of ferroptosis through integrin αvβ3/Hippo signaling all support mechanistic coupling between load and iron-dependent death (Xiang et al., 2024; Ke et al., 2023b; Shi et al., 2026; Wang et al., 2026). CEP cells may also enter ferroptosis through YAP/TEAD1/NCOA4 (Wang et al., 2025). However, direct comparison across compartments and multi-laboratory replication are lacking, and ferroptosis remains an emerging rather than an established organizing principle. PANoptosis has been reported in AF cells (Zheng et al., 2025), and bioinformatic studies have generated hypotheses regarding necroptosis and disulfidptosis, but these remain at the computational prediction stage (Lv et al., 2025).

### 4.5 Mitochondrial stress as a shared platform

Mitochondria are now recognized as direct readers of mechanical state in disc cells (Ke et al., 2023a; Gu et al., 2025; Madhu et al., 2023). Based on these findings, a working model of the mechanically driven mitochondrial stress cascade can be proposed, although the causal ordering of its steps requires further validation (Ke et al., 2023a; Gu et al., 2025; Xu et al., 2025; Madhu et al., 2023; Jia C. et al., 2024; Zhai et al., 2026). In NP cells exposed to matrix stiffening, the Piezo1/Drp1 arm drives pathological mitochondrial fission and reduces oxidative phosphorylation capacity (Ke et al., 2023a). The SPP1/integrin α5β1 axis blocks mitophagy in calcifying NP cells by inhibiting PINK1/PARKIN-dependent ubiquitination (Gu et al., 2025). Selenium supplementation protects NP cells from overloading-induced ferroptosis partly by preserving mitochondrial GPX4 function (Jia C. et al., 2024). Rejuvenation of THY1+ NP cell-derived stem cells through the FGF10/FGFR1/CREB pathway also converges on mitochondrial fission (Zhai et al., 2026). Matrix stiffness further drives glycolytic reprogramming through MRTF-A, and BNIP3 has been implicated in NP mitochondrial homeostasis and metabolic regulation (Xu et al., 2025; Madhu et al., 2023). Circadian disruption may further gate this stress platform by coupling PER2-mTOR, autophagy, and mitochondrial quality-control programs (Yuan et al., 2025; Ding et al., 2021).

Collectively, mitochondrial fission, mitophagy failure, metabolic reprogramming, and increased ROS appear to form a shared stress platform downstream of several mechanosensory inputs. The plausible temporal sequence begins with Ca^2+^ influx through mechanosensitive channels, progresses through Drp1-mediated fission and ROS generation, and culminates in mitophagy failure that tips the cell toward either ferroptosis or pyroptosis depending on iron availability and inflammasome priming. This model is consistent with available data (Ke et al., 2023a; Gu et al., 2025; Xu et al., 2025; Madhu et al., 2023; Jia C. et al., 2024; Zhai et al., 2026) but has not been directly tested as a complete sequence in any single intervertebral disc system.

## 5 Extracellular matrix remodeling as a closed-loop endpoint

The ECM is both the mechanical substrate of mechanotransduction and one of its principal outputs, giving the disc a closed-loop architecture in which every mechanical perturbation alters the very substrate that will be sensed next (Figure 4) (Zhang S. et al., 2022). The dominant catabolic effectors are the MMP family (MMP-1, MMP-3, MMP-15) and ADAMTS-4/ADAMTS-5, which degrade aggrecan and the collagen II network (Zhang S. et al., 2022). The dominant anabolic effectors include collagen II, aggrecan, and LOX-associated collagen crosslinking (Zhao et al., 2022; Zhang S. et al., 2022). Mechanical control of this catabolic-anabolic balance operates through the downstream arms described above, including YAP/TEAD under matrix stiffness (Zhou et al., 2023; Totaro et al., 2018), NF-κB/MAPK downstream of Piezo1 and integrin β1, and PKC-associated matrix-remodeling responses (Zhao et al., 2022; Quan and Kim, 2024). Hydrostatic-pressure regimes that mimic the diurnal spinal loading rhythm maintain anabolic turnover in bovine NP cells, and deviation from this rhythm tips the balance toward catabolism (Vieira et al., 2021). Engineered hydrogel studies further suggest that reduced matrix viscous dissipation may contribute to degeneration and may be partially rescued through biomaterial design (Yu et al., 2026). Perfusion combined with dynamic mechanical loading is now being incorporated into *in vitro* models of the AF/CEP interface, enabling more direct study of boundary-zone remodeling under controlled loading (Thom et al., 2026). Shear stress can also directly regulate ECM and MMP gene expression in human AF cells, supporting AF-specific boundary-zone mechanobiology (Chou et al., 2016). The research profile is strongest in preclinical systems for pathological-load-induced MMP and ADAMTS upregulation across *in vitro*, organ-culture, and rodent studies, whereas quantitative dose-response relationships in human tissue remain absent.

## 6 Experimental platforms and multiscale technologies

**FIGURE 5:**
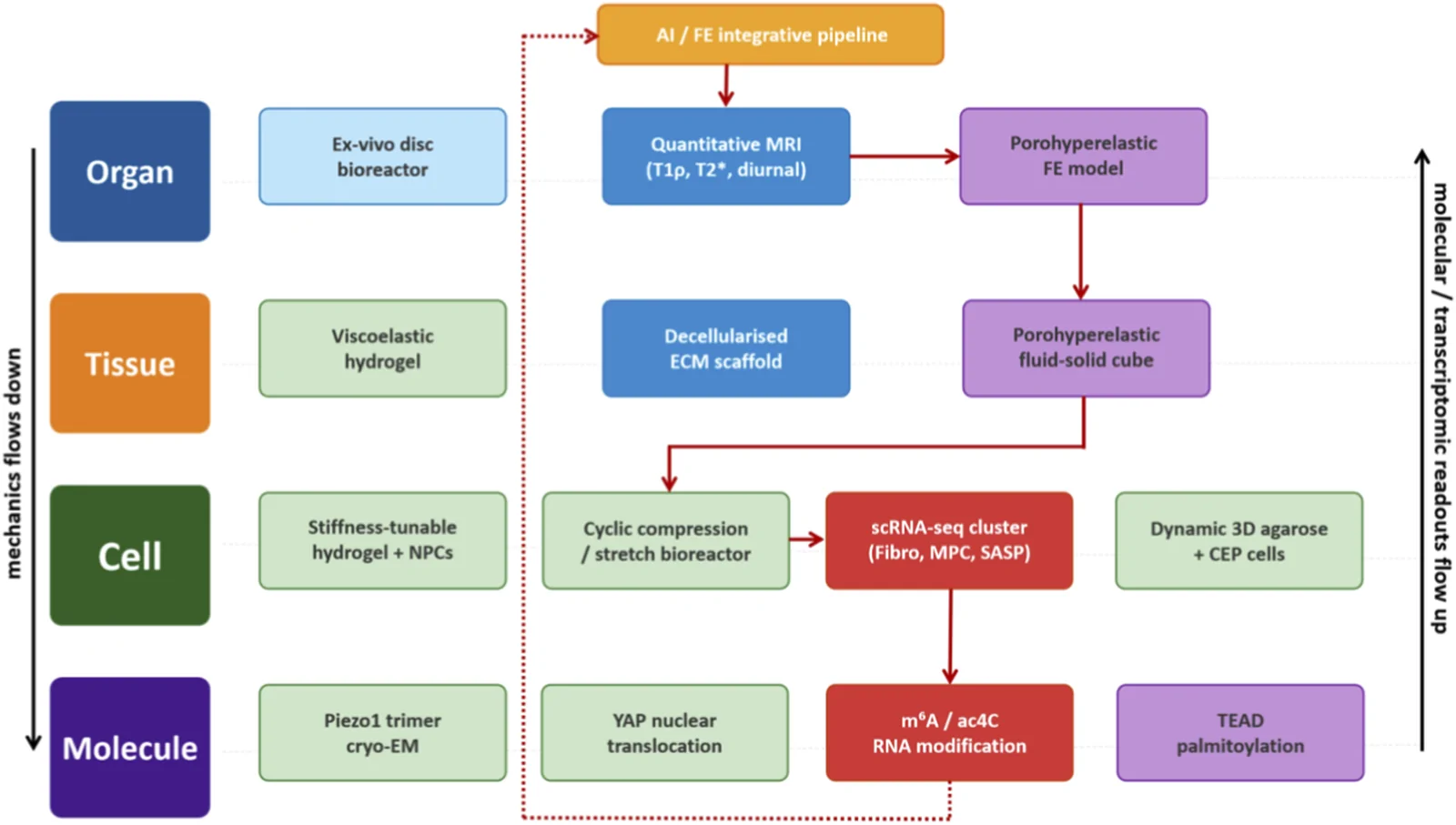
Multiscale mechanobiology research framework. This diagram outlines a hierarchical framework spanning organ, tissue, cellular, and molecular levels, illustrating how mechanical forces flow downward while molecular and transcriptomic readouts feed back upward. It integrates diverse methodologies across scales, from macroscopic quantitative MRI and porohyperelastic FE models to tissue-level fluid-solid modeling, cyclic compression/stretch bioreactors, scRNA-seq clustering, and molecular assays. Solid red arrows denote model-informed mechanical prediction and experimental validation across scales, whereas the dashed red arrow denotes molecular/transcriptomic feedback to the AI/FE integrative pipeline for model refinement and hypothesis prioritization.

The disc spans organ, tissue, cell, and molecular length scales, and no single experimental platform is sufficient to address the full range of mechanobiological questions (Figure 5). The relevant criterion for evaluating each platform is which mechanistic gap it can close.

Complex compression-torsion paradigms combined with pro-inflammatory cytokine exposure are beginning to define the inflammatory loading conditions that deserve direct biological testing across compartments (Crump et al., 2026). In parallel, finite element (FE) modeling and magnetic resonance imaging (MRI)-informed biomechanical pipelines now capture compartment-specific loading, geometry, and stress distributions across the motion segment, and their key value lies in defining mechanical states that can subsequently be tested experimentally (Hess et al., 2023). FE and data-integration approaches are most informative when they narrow the mechanical conditions that deserve direct biological testing across compartments, rather than serving as standalone predictive tools.

Hydrogels with tunable stiffness and viscoelasticity provide the most direct causal platform for mechanotransduction studies at the cell level, because they isolate one physical variable at a time (Blache et al., 2022; Zhang et al., 2025). Their main value lies in enabling receptor-level and pathway-level comparisons under controlled boundary conditions. Hydrogel-to-cell composites extend this logic toward repair, with glycoengineered stem-cell hydrogels, ion-optimized nanoparticle systems, autologous NP cell hydrogels, and antioxidant carriers demonstrating how mechanical tuning, cell survival, and biochemical support can be combined (Li et al., 2025d; Wang Z. et al., 2023; Rosenzweig et al., 2018; Wang X. et al., 2024). A recent viscoelastic hydrogel study linked restoration of disc mechanical homeostasis to YAP pathway modulation (Yu et al., 2025). Laminin-mimetic peptide hydrogels preserve a healthy NP cell gene-expression signature and oppose fibrotic drift, reinforcing the view that matrix mechanics and biochemistry are active determinants of NP identity rather than passive context (Speer et al., 2021). Broader tissue-engineering strategies that combine seed cells, scaffolds, and bioactive factors for disc regeneration have recently been reviewed (Nie et al., 2025).

For CEP and AF questions specifically, 3D agarose compression systems and hydrostatic-pressure plus deviatoric-strain models are valuable because they move the field beyond NP-focused stiffness screens and begin to resolve compartment-specific responses (Crump et al., 2025; Taiji et al., 2024). Organ culture occupies the bridge between isolated-cell causality and whole-animal complexity, preserving tissue architecture, the AF/CEP interface, and load transfer across the motion segment while remaining experimentally controllable (Thom et al., 2026; Rosenzweig et al., 2018; Kanan et al., 2022). It is therefore especially suited for testing whether NP-derived candidate mechanisms persist when compartment coupling is preserved.

Quantitative MRI provides the matching *in vivo* readout. Diurnal T1-rho variation, *in vivo* fluid-transport mapping, and MRI-informed biomechanical models connect molecular hypotheses to measurable human physiology (Hess et al., 2023; Martin et al., 2022; Hamaguchi et al., 2023). In practical terms, MRI is most useful when it filters which mechanobiology hypotheses are clinically plausible and identifies which compartments most warrant matched experimental comparison. Data integration remains comparatively immature. Most artificial intelligence (AI) or machine-learning work in IVDD still concerns image-based diagnosis, segmentation, or model construction rather than direct mechanistic inference (Hess et al., 2023). The near-term value of data integration lies in prioritizing patient-specific biomechanical scenarios and nominating cross-compartment questions that warrant experimental testing. These read-outs could also help stratify patients by their dominant degenerative phenotype and identify those most likely to benefit from mechanobiology-targeted therapy (Hess et al., 2023; Martin et al., 2022; Hamaguchi et al., 2023). Patient-specific finite-element models built from individual imaging could further localize compartment overload and personalize load modification (Hess et al., 2023; Martin et al., 2022; Hamaguchi et al., 2023). A practical translational pathway would therefore use quantitative MRI and finite-element modeling to define the dominant mechanical phenotype, select patients for load modification, local biomaterial repair, or molecular targeting, and then monitor whether the chosen intervention restores a safer compartment-specific mechanical window.

Computational systems biology complements these platforms. Regulatory-network and multiscale models of nucleus pulposus cells now reproduce anabolic and catabolic states and couple microenvironmental inputs to tissue-level behavior (Tseranidou et al., 2025; Baum et al., 2021), offering a route to rank mechanosensor hierarchy, resolve temporal ordering, and compare compartments *in silico* before experimental validation. Future frameworks could connect scRNA-seq-derived cell states, proteomic or epigenetic readouts, and MRI-to-FE stress maps so that predicted local loads can be tested against compartment-specific molecular responses (Hess et al., 2023; Martin et al., 2022; Hamaguchi et al., 2023; Tseranidou et al., 2025; Baum et al., 2021). Such models would also allow candidate sensor hierarchies to be stress-tested across NP, AF, CEP, and interface contexts before organ-culture or animal validation.

## 7 Translational landscape and therapeutic implications

**FIGURE 6:**
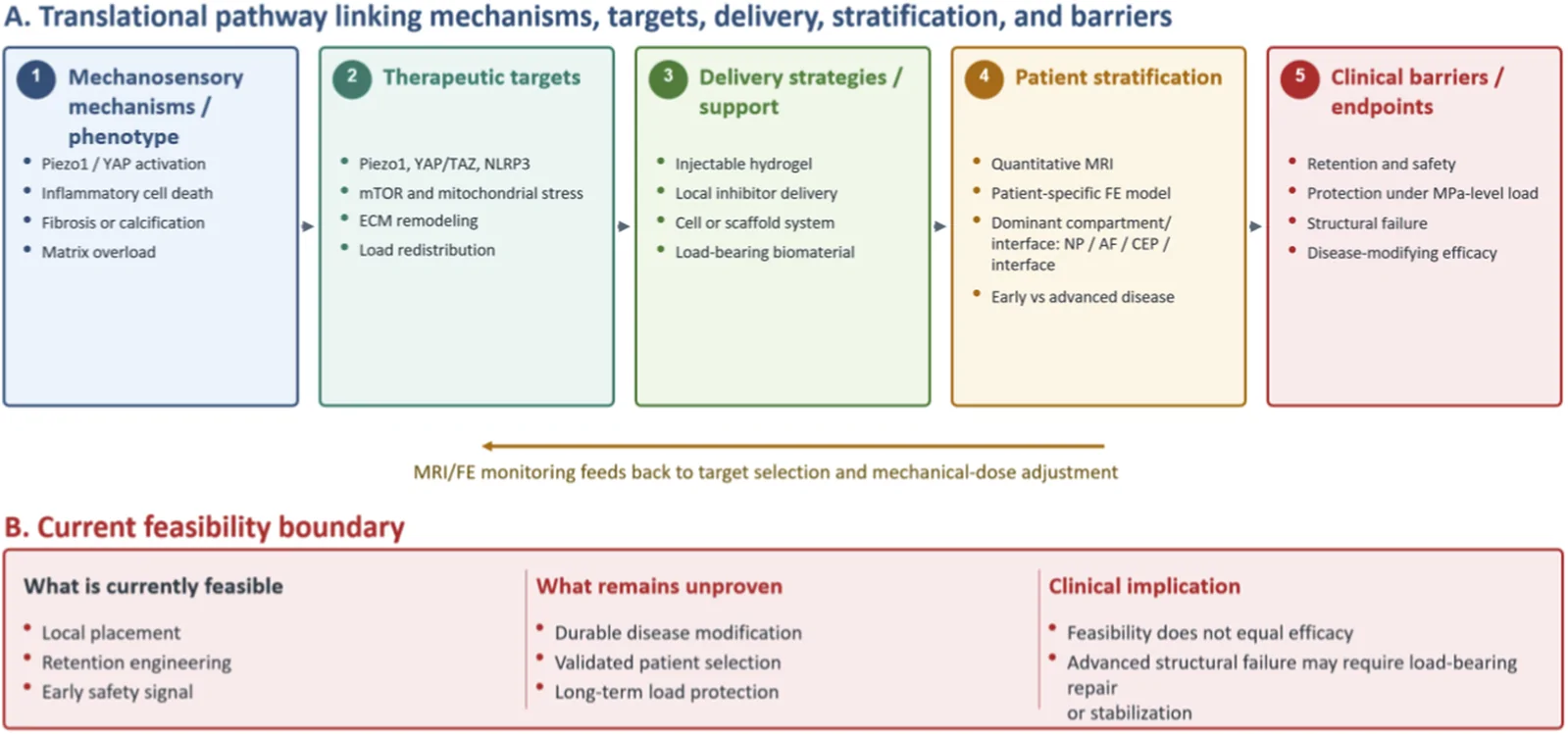
Translational pathway and feasibility boundaries of mechanotargeting in IVDD. **(A)** Proposed pathway linking mechanosensory mechanisms and degenerative phenotypes to therapeutic targets, delivery or mechanical-support strategies, patient stratification, and clinical barriers/endpoints. **(B)** Current feasibility boundary showing that local delivery, retention, and early safety signals do not yet establish durable disease-modifying efficacy, especially under advanced structural failure or MPa-level physiological loading.

Therapeutic development in IVDD remains constrained by a gap between mechanistic richness and clinical translation (Figure 6) (Zhang et al., 2025; Kumar et al., 2025). Most mechanobiology-informed IVDD interventions remain at an early translational stage, with work still largely confined to *in vitro*, *ex vivo*, or animal models and progressing through target engagement, preclinical validation, human feasibility, and clinical efficacy.

Physical interventions that act through the disc-endplate-vertebral axis rather than through disc cells alone merit particular attention. Studies of endplate-mediated nutrient transport, vertebral endplate vascular budding under axial compression-distraction, and motion-segment stress redistribution each support a mechanobiological rationale for load modification (Zhan et al., 2021; Giers et al., 2017; Han et al., 2025). These studies do not yet define a validated disease-modifying prescription. Andrographolide protects NP cells from mechanical-pressure-induced autophagy dysregulation and oxidative stress via a miR-9/FoxO3/PINK1/Parkin axis, providing a natural-product approach that targets mechanically relevant mitophagy machinery (Liu et al., 2024). Evidence from developmental biology further underscores the depth of mechano-dependence, with prenatal muscle forces being necessary for normal vertebral segmentation and disc structure in mice (Levillain et al., 2021).

Candidate molecular interventions remain at the preclinical stage. Piezo1 blockade has rescued ferroptosis, mitochondrial fragmentation, senescence signaling, and autophagy/apoptosis imbalance in cell and *ex vivo* systems (Shi et al., 2022; Xiang et al., 2024; Wu et al., 2022; Ke et al., 2023a), and disc-specific Piezo1 deletion in mice supports biological plausibility (Li F. et al., 2025). Four caveats currently limit any Piezo1-directed program. First, Piezo1 is essential in vascular, hematologic, bone, and cartilage biology, making local rather than systemic delivery likely mandatory. Second, existing tools such as GsMTx4, Verteporfin, and MCC950 have selectivity, retention, or off-target limitations. Third, compartment-specific effects, especially the protective AF ossification finding, complicate uniform blockade. Fourth, no controlled human trial of Piezo1-, YAP-, or NLRP3-directed IVDD therapy has been reported (Shitozawa et al., 2026).

Biomaterial delivery currently offers one of the clearest bridges between mechanobiology and human feasibility, because biomaterials alter tissue-level mechanics while accommodating local delivery (Zhang et al., 2025). IL-33-targeting hydrogels, NP-matched viscoelastic injectables, antioxidant carriers, and cell-supportive matrices (Li et al., 2025d; Rosenzweig et al., 2018; Wang X. et al., 2024; Speer et al., 2021; Zheng et al., 2026; Jia et al., 2022) all operate on the principle that changing the mechanical context can modify downstream biology. An early sign that biomaterial mechanobiology may approach clinical translation came in 2025, when a single-arm, 1-year feasibility study of an injectable hydrogel implant for painful lumbar degenerative disc disease reported acceptable safety and early functional improvement (Clerk-Lamalice et al., 2025). This trial is small and uncontrolled, but it demonstrates that a mechanically tuned injectable can reach patients and provides a preliminary benchmark for subsequent iterations.

Three persistent bottlenecks constrain clinical translation. First, patient stratification remains weak, with no validated biomarker currently identifying patients whose disease is predominantly driven by mechanosensor-related, ferroptotic, or calcific processes (Hess et al., 2023; Zhang et al., 2025; Kumar et al., 2025; Clerk-Lamalice et al., 2025). Second, the therapeutic window is narrow, as successful rescue studies almost always target early-to-moderate degeneration rather than advanced disease. Third, the mechanical-dose question remains unresolved, because the boundary between therapeutic and pathological loading is likely compartment- and patient-specific. Patient-specific biomechanical modeling and loading simulations are promising but not yet integrated with intervention studies (Hess et al., 2023; Han et al., 2025). The megapascal-level loads on the lumbar disc can also cause structural failure such as endplate fracture, which lies beyond the reach of cellular mechanotransduction (Brinckmann et al., 1988). Regenerative therapies will therefore likely need mechanically competent, load-bearing biomaterials or added stabilization to protect repair under physiological load *in vivo* (Zhang et al., 2025; Yu et al., 2025; Jia et al., 2022; Brinckmann et al., 1988). In advanced degeneration, this mechanical-protection window may become more important than single-pathway inhibition, because collapsed endplates, annular rupture, or gross instability are unlikely to be reversed by molecular mechanotargeting alone (Qiao et al., 2026; Osti et al., 2025; Zhang et al., 2025; Brinckmann et al., 1988).

## 8 Emerging frontiers and future directions

Several unresolved issues still constrain the field’s shift from descriptive expansion to comparative explanation, highlighting the need for matched comparisons across disc compartments and interfaces. The disc increasingly appears to function as a peripheral circadian organ, with bidirectional interactions between mechanical loading and intrinsic clock rhythms (Yuan et al., 2025; Ding et al., 2021; Hamaguchi et al., 2023; Chen J. et al., 2026; Peng et al., 2022; Zhou et al., 2024; Sang et al., 2025). Excessive strain dampens NP rhythmicity, circadian disruption accelerates degeneration, and clock-regulated factors such as Nrf2, Rev-erbα, and BMAL1 influence inflammatory and mitochondrial outputs (Yuan et al., 2025; Ding et al., 2021; Peng et al., 2022; Zhou et al., 2024; Sang et al., 2025). These observations support phase-resolved mechanobiology experiments, although clock-directed interventions, including BMAL1-related strategies with EGCG and melatonin, remain confined to cell and animal studies (Kaye et al., 2024; Mei et al., 2021; Lei et al., 2025; Migliorini et al., 2026). Mechanical memory extends this theme beyond circadian timing, and emerging work on non-coding RNAs, m6A regulation, lactylation, and extracellular-vesicle signaling suggests that epigenetic regulation also contributes to mechanotransduction, although its dominant and most tractable layers remain unclear (Yang et al., 2021; Sun et al., 2025; Sheng et al., 2024; Li L. et al., 2026). At the opposite extreme of loading, spaceflight and related models are linked to disc swelling and a higher risk of herniation after return to gravity (Johnston et al., 2010; Treffel et al., 2020). Together, these findings suggest that too little loading may also be harmful and support a mechanical set-point view of disc homeostasis, in which unloading-related swelling or altered matrix hydration and subsequent reloading are considered paired mechanical challenges (Johnston et al., 2010; Treffel et al., 2020). These clock interactions feed back onto YAP/TAZ, mTOR, mitochondrial quality control, autophagy, inflammation, and matrix turnover, so the timing of loading matters as much as its magnitude (Yuan et al., 2025; Ding et al., 2021). A recent systems-level review frames these bidirectional mechanotransduction and circadian interactions across the intervertebral disc and articular cartilage (Workineh et al., 2026).

At the mechanosensor level, disc research remains overwhelmingly Piezo1-focused, with no comparable body of Piezo2-directed work in NP, AF, or CEP cells. Current Piezo-mediated claims in the disc therefore refer primarily to Piezo1, while the absence of parallel Piezo2 data is better viewed as a research gap than as an indication of irrelevance (Ranade et al., 2014). More broadly, data integration remains prospective rather than mature. The MRI-to-FE pipeline is the clearest example, but coupling local stress maps with transcriptomic or proteomic outputs is still not established (Hess et al., 2023). Mechanobiology-based therapy will also require mechanobiology-based patient stratification, yet current single-cell and transcriptomic findings have not translated into patient selection. Regenerative strategies, including stem and progenitor cell mechanobiology, THY1+ NP cell rejuvenation, and mechanically tuned biomaterials, remain preclinical (Zhai et al., 2026; Wang Z. et al., 2023; Jia et al., 2022; Zhao et al., 2025). Progress will also depend on next-generation models with greater compartmental, temporal, and boundary-condition resolution, since most current *in vivo* systems underrepresent the coupled disc-endplate-vertebral environment and existing genetic models do not adequately separate NP, AF, and CEP contributions (Kanda et al., 2021; Osti et al., 2025; Hu Z. et al., 2024; Ding et al., 2021).

## 9 Discussion

Mechanotransduction in IVDD has progressed from a descriptive link between mechanical load and disc degeneration to a molecular framework with identifiable sensors, downstream signaling networks, and cell-fate outputs (Qiao et al., 2026; Ke et al., 2025). Piezo1 is the most intensively studied branch, with support from human NP cell studies, rodent conditional knockout models, and multiple downstream effector pathways (Guo et al., 2023; Li F. et al., 2025). Even so, a definitive hierarchy has not yet been established. The concentration of mechanistic data in NP models, together with the near-absence of matched cross-compartment comparisons, means that the relative importance of Piezo1 versus TRPV4, integrins, ASICs, and primary cilia in AF, CEP, and interface contexts remains unresolved.

Three conclusions can nevertheless be drawn with reasonable confidence. First, the disc contains a diverse mechanosensory repertoire that converges on a smaller set of shared downstream hubs, most notably YAP/TAZ and MAPK/NF-κB, which then diverge into compartment-weighted cell-fate outcomes including senescence, pyroptosis, and ferroptosis. Second, the ECM functions as both an input and an output of mechanotransduction, creating a closed-loop architecture in which degenerative changes in matrix stiffness and composition progressively amplify pathological mechanosensing (Kurakawa et al., 2015; Zhang S. et al., 2022; Blache et al., 2022). Third, therapeutic translation remains at an early stage, with the clearest progress coming from biomaterial-based strategies that simultaneously modify tissue mechanics and enable local delivery (Zhang et al., 2025; Desai et al., 2024). Read together, these branches form a single sensor-to-matrix axis, summarized in Figure 4, that links mechanosensors, shared hubs, cell-fate programs, and matrix remodeling to clinical disc degeneration (Yuan et al., 2025; Wu et al., 2022; Ke et al., 2023a; Sun et al., 2026; Kurakawa et al., 2015; Song et al., 2022; Wang H. et al., 2023).

Several unresolved questions should guide future priorities. The compartment-specificity of Piezo1 function, particularly the apparently protective role of Piezo1 in AF ossification (Shitozawa et al., 2026), suggests that a simple blockade strategy may be inadequate. The lack of Piezo2 data in disc cells remains a conspicuous gap. The causal ordering within the mitochondrial stress cascade, from Ca^2+^ influx through fission, ROS accumulation, and mitophagy failure to cell death, has more often been inferred than directly tested. Chrono-mechanobiology and mechanical memory are also conceptually compelling but remain only lightly developed experimentally.

A comparative approach may therefore be especially productive. Matched cross-compartment studies using standardized mechanical stimuli and readouts, stronger AF-, CEP-, and interface-aware models, and preclinical therapeutic strategies that address local delivery, endplate transport, and vertebral boundary conditions may help translate the molecular complexity outlined in this Review into disease-modifying interventions. The field is well positioned for further progress, and that progress will likely benefit from integrative, interface-aware experimental designs that extend beyond the single-compartment, single-sensor studies that have driven the field thus far.
