# Correction: Mechanotransduction in intervertebral disc degeneration: from compartment-specific sensors to translational frontiers

**DOI:** 10.3389/fbioe.2026.1953445

**Published:** 2026-08-26

**Authors:** Hongtao Li, Changxiao Han, Guangyi Yang, Jiacheng Zheng, Bochen Peng, Jiali Chen, Hanbin Liu, Liguo Zhu, Minshan Feng

**Affiliations:** 1 Department of Spine Surgery, Wangjing Hospital, China Academy of Chinese Medical Sciences, Beijing, China; 2 Beijing Key Laboratory of Digital Intelligence Traditional Chinese Medicine for Preventing and Treating Degenerative Bone and Joint Diseases, Beijing, China

**Keywords:** cartilage endplate, intervertebral disc degeneration, mechanosensors, mechanotransduction, nucleus pulposus, Piezo1

There was a mistake in Figure 1B as published. The annulus fibrosus was depicted as extending beneath nearly the entire cartilage endplate, thereby obscuring the direct interface between the central nucleus pulposus and the cartilage endplate. Anatomically, the cartilage endplate overlies the nucleus pulposus centrally, whereas the annulus fibrosus contacts the more peripheral portions of the endplate. The corrected Figure 1 appears below. This correction is limited to the schematic representation and does not affect the conclusions of the article.

**FIGURE 1:**
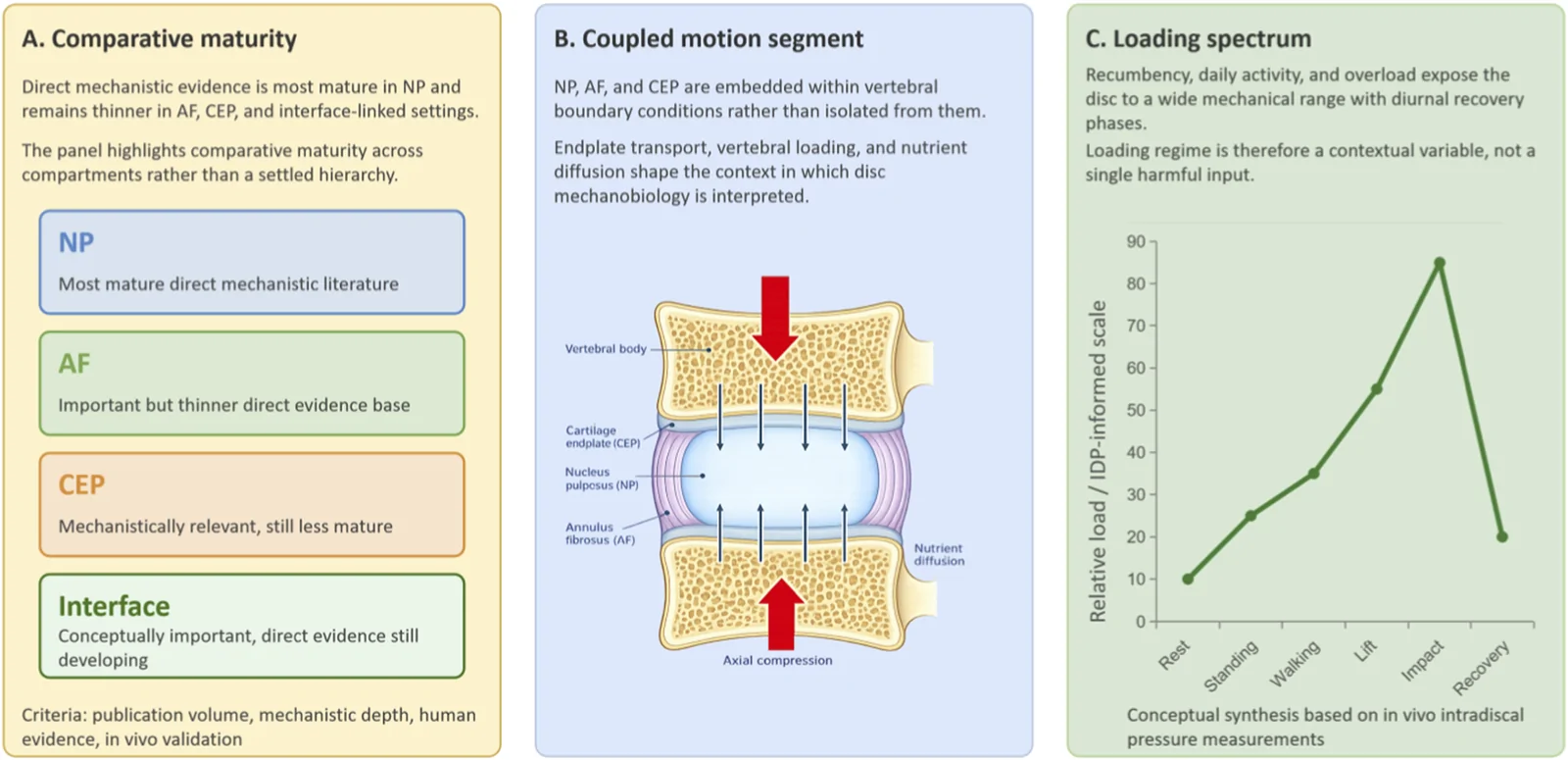
NP-predominant mechanobiology in IVDD within a disc-endplate-vertebral interface framework. **(A)** Comparative evidence maturity across NP, AF, CEP, and interface-associated mechanobiology, qualitatively ranked according to publication volume, mechanistic depth, human-tissue support, and *in vivo* validation. **(B)** Coupled motion-segment context showing that NP, AF, and CEP are mechanically and biologically constrained by vertebral boundary conditions, endplate transport, and nutrient diffusion. **(C)** Conceptual loading spectrum across recumbency, daily activity, and overload/recovery states, synthesized from reported *in vivo* intradiscal pressure measurements ranging from approximately 0.1 MPa during recumbency to above 1 MPa during upright activity and above 2 MPa during lifting (Wilke et al., 1999).

The original article has been updated.

